# Parenting children with autism spectrum disorder in the United Arab Emirates: Exploring perceptions towards using the picture exchange communication system to enhance the development of children with autism spectrum disorder

**DOI:** 10.1186/s40359-022-00932-3

**Published:** 2022-09-30

**Authors:** Mohammed F. Safi, Mariam Alshamsi, Maxwell Peprah Opoku

**Affiliations:** 1 Speech Language Pathology College of Medicine and Health Sciences, United Arab Emirates University, Al-Ain, United Arab Emirates; 2 Special Education Department, United Arab Emirates University, Al-Ain, P. O. Box 15551, United Arab Emirates

**Keywords:** Autism spectrum disorder, PECS, Developmental disability, Communication, Early intervention, United Arab Emirates, Parents

## Abstract

**Background:**

Autism spectrum disorder (ASD) is an interplay between biological, social and environmental factors that impact the development of individuals. However, core characteristics are social, communication and behaviour challenges that restrict children’s participation in society. Consequently, there are discussions regarding the need for society to develop interventions that are geared towards promoting the participation of children with ASD in societies. While the Picture Exchange Communication System (PECS) helps lessen the biological characteristics of children with ASD, its impact has rarely been explored in non-western societies, such as the United Arab Emirates (UAE).

**Objectives:**

The instant study’s purpose was to explore parents’ perceptions of the effectiveness of the PECS on the communicative, social and academic developments of children with ASD in UAE.

**Method:**

The Perception Towards Picture Exchange Communication Scale (PTPECS) was developed to collect data from the participants. The newly developed tool was piloted and validated before being implemented. The participants included 73 caregivers of children with ASD in the UAE.

**Results:**

The following non-parametric tests were conducted: Mann-Whitney U test, Kruskal-Wallis test, and spearman rho. The results revealed that the parents were generally positive about employing the PECS to support the development of their children with ASD. There was also a positive association between using the PECS and improving communication, learning and social skills in children with ASD.

**Conclusion:**

It is imperative that educators and policymakers envisage parents as equal partners in providing rehabilitation services to children with ASD. Various implications, such as the need for continual engagement and empowering parents of children with ASD, are discussed.

## Background

Autism spectrum disorder (ASD) is an interplay between biology, social context and environmental factors that may impact one’s development [1]. The neurodiversity paradigm acknowledges the biological understanding of ASD as being characterised by individual differences in three major domains: social, language and repetitive behavioural patterns [2–4]. Specifically, individuals diagnosed with ASD face challenges in areas of social skills and verbal and nonverbal communication [5–8], leading to language delay and learning difficulties [3, 4]. However, as heterogeneous disorders, not all of the symptoms of ASDs are manifested in all patients or children, and many symptoms are also common in other disorders [2, 3, 8]. This underscores the need for the context within which individuals with ASD are raised put mechanisms in place to enable them to participate equally in society [1]. This is placed against the backdrop of the fact that the number of children with ASD is rising and a sizeable number are living in society [4]. For example, there are prevalence rates of 1.5% in the United States [9], 1.1% in Europe, 1.9% in Asia, 2.4% in the Middle East [10] and 0.29% in the United Arab Emirates (UAE) [11]. It is estimated that between 25% and 30% of children with ASD and 50% of adults with ASD may never develop spoken language [12]. Accordingly, proponents of the social model of disability [1] have suggested the need for society to develop effective interventions that address the relevant characteristics, such as communication delays, and social and behavioural problems [13, 14] that restrict the participation of children with ASD in society.

The challenges that children with ASD face in society cannot be overemphasised [3, 9–12]. Many scholars have recommended approaches to support the development of social communication and learning skills of children with ASD [15–17]. These interventions include augmentative and alternative communication (AAC) [17], human-computer interface [17], interactive smart environments [18], virtual voice assistants to increase communication abilities [18], social stories to enhance social skills [16] and trained dogs and meditation [19] to lessen the ASD symptoms. Augmentative and alternative communication is utilised to assist individuals with ASD with their communication and speech [15, 20], and thus improve their social communication. Its approaches may include unaided, as well as aided, systems. While the former uses gestures and sign language, the latter utilises speech-generating devices, writing boards and/or pictures to communicate. Professionals use aided AAC approaches to enhance communication abilities and social interaction, and address learning difficulties in children with ASD [21].

The Picture Exchange Communication System (PECS) is one of the most common aided AACs employed to overcome fine motor problems that make learning through signs and symbols more difficult [15]. The PECS is a form of applied behaviour analysis (ABA) that is widely used to improve core deficits associated with ASD [21]. In general, several studies have found AAC approaches were perceived as important, effective and acceptable [22–27]. However, the field of ABA is not without controversies as there are discussions on appropriate ways to implement interventions that do not violate the fundamental rights of children with ASD. Specifically, rights-based groups have critiqued punishment-based procedures and have advocated for a more child-friendly intervention to enhance the well-being of children with ASD [21].

The PECS, which Bondy and Frost [28, 29] developed in 1985, was designed to teach children with ASD how to engage in spontaneous communication by considering the unique characteristics of children with autism, including their restricted motor and verbal imitation skills, lack of attending skills and insensitivity to social rewards [7, 28, 29], as well as the need to integrate them into society [30]. Since at least two people are needed to communicate, children with ASD observe a setting where two people are communicating in an effort to teach them how to communicate. Subsequently, words are linked to pictures. The PECS is thought to differ from other communication systems in that prerequisite skills are not needed. Furthermore, it was designed to address the lack of motivation for social reinforcement and teaches immediate initiating rather than responding before initiating [7].

A plethora of literature has revealed that the PECS is an effective and appropriate approach to developing functional communication abilities, social interactions and learning skills in individuals with ASD [28, 29, 31–37]. Tien [37], by conducting a research synthesis that included 13 studies and 125 participants with ASD who had limited or no functional communication skills, revealed that the PECS was effective as a communication tool; enhanced one’s level of communication and language; increased spontaneous language, speech, and/or imitations; improved communication initiations; and increased the mean length of utterances [38–40]. Charlop-Christy et al. [38], in a study that implemented a single-subject multiple baseline design, demonstrated that older children increasingly used pictures to make requests and also increased their spontaneous production of spoken requests. Furthermore, a case study of a three-year-old boy found that the PECS induced verbal demands and other initiations in both the home and kindergarten, generalized settings, as well as increases in spoken vocabulary and the length of comprehensible spoken utterances during free play [32]. A systematic review study concluded that the PECS is a promising, but not yet established, evidence-based intervention to facilitate communication in children with ASD between the ages of one and 11 years, and further revealed that small to moderate gains in communication were demonstrated following training [7]. Howlin et al. [31], who investigated the effectiveness of classroom usage of the PECS in 84 children with ASD, found that those children whose teachers, parents and staff received PECS training-initiated communication at a higher rate when using the PECS. The PECS’ effects on spoken language have been shown to be small to negative [31, 34, 36]. While the advantages of the PECS have been revealed in western countries, only a scarce amount of research has explored the impact of the PECS on children’s development in non-western countries.

In this study, the terms “parents” and “caregivers” are used interchangeably. It is noteworthy that only a few studies have been conducted on parental perspectives of the impact of PECS on the lives of their children with ASD. Alsayedhassan et al. [41] explored parental knowledge and barriers to using PECS among their children with ASD in the United States. Although the parents reported being knowledgeable and having experienced ease with their children using PECS in their daily lives, they acknowledged notable barriers to its use on a daily basis. Alsayedhassan et al. [41] also reported notable differences between the participants. They found that the higher the parents’ income, the more knowledgeable they were, and the less likely they were to report barriers when using the PECS to support their children. Furthermore, the more educated the parents, the more knowledgeable they were about the PECS. However, Alsayedhassan et al. [41] failed to address parents’ perceptions of the domains that individuals with ASD appeared to have difficulty with in their daily lives. It is evident, for example, that children with ASD struggle in their daily communication, learning and relating to others [2, 3]. Bondy and Frost [28, 29] focused on the PECS’ ability to enhance communication, improve academic skills and foster positive behaviour in children with ASD. Consequently, it is imperative that studies on the impact of PECS should address the important domains or relevant to the development of children with ASD [21]. Accordingly, the purpose of the present study was to explore parents’ and primary caregivers’ perceptions of the use of the PECS to promote the development of children with ASD in the UAE. This study promises to add to the literature a standardised measure that could be used to measure the impact of ABA intervention [21], such as PECS on the development of children with ASD in a non-western context.

## Rationale for this study

The role of parents in the development of children with developmental disabilities such as ASD cannot be overemphasised [3, 8]. Specifically, due to the functional and activity limitations associated with ASD [1–3, 5], it is crucial that parents act on their behalf and support their development at home [41]. The parents’ roles have been acknowledged throughout the world, and various laws related to their importance have been created [42]. The United Nations, through the promulgation of the Convention on the Rights of Persons with Disabilities, made provisions for parents to play a formidable role in rehabilitating their children with ASD [42]. In the UAE, laws such as Federal Law Number 29 of 2006 [44], Federal Decree number 116 of 2009 [45], and Law number 2 of 2014 [46], each advanced to promote the social inclusion of individuals with disabilities, have reiterated the need for parents to take a leading role in choosing appropriate intervention services and supporting the optimal development of their children at home.

The search is ongoing for effective approaches to realising an inclusive environment for all in the UAE. Accordingly, the perspectives of the primary caregivers of children with ASD are beneficial for guiding policies and rehabilitation studies. However, there is a lack of data on caregivers’ perceptions of the use of interventions, such as the PECS, to help children with ASD develop. Accordingly, this study explored parents’ perceptions of the effectiveness that PECS has on the development of their children with ASD. It appears that this study is the first of its kind, with the potential to inform policy on the enhanced development of children with ASD. It was guided by the following research questions:

- What are parents’ perceptions of the use of the PECS among children with ASD in the UAE?
- What is the association between parents’ background variables and perceptions of the use of the PECS among children with ASD in the UAE?
- Will the use of the PECS enhance the communication, learning and social interaction skills of children with ASD in the UAE, as perceived by parents?

## Materials and methods

### Study participants

The participants included caregivers of children with ASD in the UAE. The participants were recruited from the Emirates of Abu Dhabi, Dubai and Sharjah, and by employing convenience sampling and determining their availability to participate in the study. Parents across the UAE received online training in the PECS and were encouraged to use the intervention method to communicate with their children at home. The training was conducted at the time when all schools and rehabilitation facilities for children with ASD were closed because of the outbreak of COVID. Since parents expected to train their children at home, the training was deemed necessary to enable them acquire some skills to support their children at home.

The training was facilitated by a psychologist with experience training others in implementation of PECS. The two-hour basic training was structured to cover all the six phases of PECS (how to communicate, distance and persistence, picture discrimination, sentence structure, attributes and language expansion, responsive requesting and commenting). Subsequently, the parents were invited to rate their children’s performance in terms of usage of PECS at home. The participants had to meet the following inclusion criteria: (a) caregiver of a child with ASD under the age of 18 years, (b) raising a child who had ASD and (c) gave consent to participate in the study.

Hundred and eighteen (118) parents with children with ASD who participated in the training were invited for this study. Out this, 73 completed the survey, representing a response rate of 62%. 85% were mothers, 8% were fathers and 7% indicated they were nannies. Furthermore, 35% supported children between 1 and 5 years of age and 43% supported those between 6 and 10 years of age. In addition, 19% had at least 11 years of experience caring for children with ASD. While 56% indicated their children were boys, 44% noted their children were girls. Moreover, 59% had used the PECS between 1 and 5 years and 41% used it for at least 6 years. Finally, 37% of the children were enrolled in inclusive schools, 25% in special schools, 19% in rehabilitation centres and 19% in early childhood intervention centres (Table1).

**Table 1:** Summary of the Mean Scores

Items	M	SD
***Communication skills***	5.09	0.57
In my opinion, the PECS helps the child with autism to express their desires and needs	5.12	0.54
Based on my experience, the PECS helps the child with autism to express their feelings	5.09	0.70
In my view, PECS stimulates and improve spoken language among children with ASD.	4.84	1.10
Based on my experience, the PECS helps the child with autism to initiate communication and respond to others.	5.05	0.79
Based on my experience (PECS) contributes to better communication skills among children with autism	5.36	0.79
***Learning skills***	5.12	0.53
In my opinion, the PECS encourages the child with autism to learn new vocabulary	5.09	0.81
Based on my experience, the PECS helps the child with autism improve their performance in academic subjects (mathematics, reading)	4.97	0.67
In my opinion, the PECS helps the child with autism learn appropriate behaviours	4.95	0.80
Based on my experience, the benefit from the PECS in education varies between individuals	5.10	0.75
Based on my experience, the PECS is effective with regard to the different learning skills of students with autism	5.48	0.74
***Social interaction skills***	5.18	0.45
In my opinion, the PECS helps the child with autism initiate interactions with peers (make new friends and play with them)	5.16	0.57
Based on my experience, the PECS helps the child with autism improve their skills to participate in group activities	5.25	0.62
Based on my perception, the PECS encourages the child with autism to improve social relationships with their parents, teachers and others	5.16	0.59
In my opinion, the amount of vocabulary used in the PECS is sufficient to complete the communication processes in various social activities	4.77	0.90
Based on my experience of the PECS, the development of social interaction skills varies greatly between individuals	5.36	0.65
In my opinion, the PECS is the best way to improve social skills in children with autism	5.41	0.76

PECS = Picture Exchange Communication System; M = Mean; SD = Standard deviation

### Instrument

A two-part questionnaire was used to collect data. The first part of the instrument focused on the participants’ demographic characteristics, including type of caregiver, gender, child’s gender and age, number of years supporting child and using the PECS, frequency of using the PECS, institution of enrolment and age that the child began using the PECS.

To the best of our knowledge, there are no scales that measure perception of the PECS in a cultural context. Accordingly, we developed a new scale to assess perception of the PECS. The Perception Towards Picture Exchange Communication Scale (PTPECS) designed for this study comprised the questionnaire’s second part. The instrument was designed in accordance with a literature review on areas of support that children with ASD require in education, such as communication, learning and social interaction [7, 15, 20, 22–27, 31, 32, 40, 47–49].

The scale was subjected to content validation by employing the Delphi approach, which involves having specialists review the draft scale [50]. The draft scale contained items in both English and Arabic and was given to three academics, three practitioners and three parents who were all proficient in both languages in order to determine whether the items on the scale were appropriate, accurate, clear and unambiguous. The initial draft included 25 items. However, following the reviewers’ recommendations, this was reduced to 19 items. The PTPECS was comprised of three factors: communication skills, learning skills and social and behavioural skills. The items were assessed on a 6-point scale, ranging from 1 (strongly disagree) to 6 (strongly agree).

The newly developed instrument was piloted among regular classroom teachers (N = 218) who taught students with autism across UAE. The teachers completed the questionnaires on a virtual platform. Thereafter, a principal components analysis using the Statistical Package for Social Science version 26 (51) was conducted on the 19 items (Table2). The correlation matrix revealed a correlation of 0.3 or more. The Kaiser-Meyer-Olkin value was 0.72, which is more than the expected 0.6. Furthermore, Bartlett’s Test of Sphericity revealed a statistical significance (p = .001), thus supporting the matrix’s factorability. Principal component analysis revealed the presence of four components with eigenvalues exceeding 1; namely, 46%, 17%, 9% and 7%. The forced fixed factor option was employed to classify the items in the three factors. The scree plot showed an apparent three breaks, thus supporting the existence of a three-factor structure. It is of interest that the negatively worded statements on the scale did not reach a coefficient value of 0.30, thus resulting in their exclusion. A total of 16 items were analysed.

**Table 2:** Summary of Factor analysis

	Items	Factor 1	Factor II	Factor III
CS_1	In my opinion, the PECS helps the child with autism to express their desires and needs	0.91		
CS_2	Based on my experience, the PECS helps the child with autism to express their feelings	0.89		
CS_3	In my view, PECS stimulates and improve spoken language among children with ASD.	0.72		
CS_4	Based on my experience, the PECS does not help the child with autism to express their emotions and desires.	0.10**		
CS_5	Based on my experience, the PECS helps the child with autism to initiate communication and respond to others.	0.82		
CS_6	Based on my experience (PECS) contributes to better communication skills among children with autism	0.71		
LS_1	In my opinion, the PECS encourages the child with autism to learn new vocabulary		0.79	
LS_2	Based on my experience, the PECS helps the child with autism improve their performance in academic subjects (mathematics, reading)		0.64	
LS_3	In my opinion, the PECS helps the child with autism learn appropriate behaviours		0.83	
LS_4	In my opinion, the PECS does not help the child with autism learn and develop new vocabulary		0.24**	
LS_5	Based on my experience, the benefit from the PECS in education varies between individuals		0.36	
LS_6	Based on my experience, the PECS is effective with regard to the different learning skills of students with autism		0.61	
SIS_1	In my opinion, the PECS helps the child with autism initiate interactions with peers (make new friends and play with them)			0.88
SIS_2	Based on my experience, the PECS helps the child with autism improve their skills to participate in group activities			0.81
SIS_3	Based on my perception, the PECS encourages the child with autism to improve social relationships with their parents, teachers, and others			0.82
SIS_4	In my opinion, the amount of vocabulary used in the PECS is sufficient to complete the communication processes in various social activities			0.32
SIS_5	Based on my experience, the PECS does not help the child with autism interact with their peers and others			0.28**
SIS_6	Based on my experience of the PECS, the development of social interaction skills varies greatly between individuals			0.54
SIS_7	In my opinion, the PECS is the best way to improve social skills in children with autism			0.44

**deleted because of factor loading below 0.03; CS/Factor I = Communication skills; LS/Factor II = Learning Skills; SIS/Factor III = Social Interaction Skills; PECS = Picture Exchange Communication System

The first sub-scale—communication skills—included five items. Examples include *In my opinion, the PECS helps the child with autism to express their desires and needs*; *Based on my experience, the PECS helps the child with autism to express their feelings*; and *In my view, the PECS stimulates the child with autism to improve their spoken language skills.*

The second sub-scale—learning skills—is comprised of five items. Examples include *Based on my experience, the PECS helps the child with autism improve their performance in academic subjects (mathematics, reading)*; *In my opinion, the PECS helps the child with autism learn appropriate behaviours*; and *In my opinion, the PECS encourages the child with autism to learn new vocabulary*.

The third sub-scale—social interaction skills—contains six items. Examples include *In my opinion, the PECS helps the child with autism initiate interactions with peers (make new friends and play with them)*; *Based on my experience, the PECS helps the child with autism improve their skills to participate in a group*; and *In my opinion, the PECS is the best way to improve social skills in children with autism*.

During the piloting stage, the scale’s internal reliability was assessed by employing Cronbach’s alpha, which yielded the following scores: overall PTPECS (0.80), communication skills (0.70), learning skills (0.72) and social interaction skills (0.71). This underscores the utility of the newly developed scale to examine perceptions of the PECS.

### Procedure

The institutional review board of United Arab Emirates University (number ERS_2021_7331) granted ethics approval. Thereafter, formal emails were sent to regular schools, rehabilitation centres for children with autism and early childhood centres that provide services to children with autism across the three Emirates of Abu Dhabi, Dubai and Sharjah. On behalf of the authors, the schools and centres contacted the parents. The research team received the details of those who agreed to participate in the PECS training. The first author sent individual emails to parents to inform them of the PECS training, subsequent study, its objectives and the benefit to the country. Prospective participants who responded positively were considered for participation. Subsequently, the prospective participants were contacted telephonically to discuss the study and provide consent to participate.

Due to the COVID 19 outbreak, the data were collected online from January 15, 2021, to April 01, 2021, using Google Forms. The invitations were sent to parents six weeks after the PECS training. The form contained both Arabic and English versions of the statements on the questionnaire. The participants were assured that their identity, as well as that of their children and the institution where their children were enrolled would remain confidential. They did not receive any reward or reimbursement for participating in the study. All participants provided oral consent when contacted to participate in this study and written, informed consent prior to completing the questionnaire.

### Data analysis

The first author transferred the data to Microsoft Excel. Subsequently, the data were transferred to Statistical Package for Social Science version 26 for analysis. Since there were less than 120 participants [51], non-parametric tests were conducted to answer the research questions. Also, a normality test using Kolmogorov-Smirnov and Shapiro-Wilk tests showed significant differences (communication, p = .005; learning, p = .001; social skills = 0.002; and total PTPECS, p = .003.) between the distributions. This connotes a violation of the assumption of normality [51] and thus, the use of non-parametric tests to answer the research questions.

To answer the first research question, the mean scores for the overall scale and sub-scales were computed. The mean scores for the participants were computed. A mean score of at least five was interpreted as a favourable perception of the PECS.

The Mann-Whitney U test and Kruskal-Wallis test were conducted in order to answer the second research question. While the Mann-Whitney U test was computed for demographic variables with two levels, the Kruskal-Wallis test was conducted for demographics with three or more levels. The magnitude of the associations between the demographic variables and parents’ perceptions were calculated using the effect size. The effect sizes were interpreted as follows: small (0.01 − 0.05), moderate (0.06 − 0.09) and large (at least 0.1) [52].

To answer the third research question, Spearman’s rho was conducted to shed light on the relationship between communication, learning and social skills. The results were interpreted as follows: small (0.1 − 0.29), moderate (0.30 − 0.49) and large (0.50 to 1) [52].

## Results

During the implementation stage, the scale’s internal consistency was assessed using Cronbach’s Alpha: PTPECS (0.90), communication skills (0.76), learning skills (0.77) and social interaction skills (0.72).

The overall mean of the participants’ perception was 5.13 (*SD* = 0.46; *Md* = 5.06). The mean scores recorded for the three sub-scales were as follows: communication skills (*M* = 5.09; *SD* = 0.57; *Md* = 5.00), learning skills (*M* = 5.12; *SD* = 0.53; *Md* = 5.00) and social interaction skills (*M* = 5.18; *SD* = 0.45; *Md* = 5.17) (Table1).

### Association between background variables and perception

A Mann-Whitney U test was conducted to assess the association between background variables and two-level demographics (years using the PECS, gender of child and frequency of using the PECS). The results showed an association between years using the PECS and the learning support sub-scale only (see Table3). Specifically, caregivers who indicated they had used the PECS between one and five years (*Md* = 5.20, *n* = 43) were more positive than those who had used the PECS for six or more years (*Md* = 5.00, *n* = 30), *U* = 454.50, *z* = -1.23, *p* = .03, *r* = .14, with a small effect size, 0.14.

**Table 3:** Summary of Mann-Whitney U test for two-level demographics

N = 73		Communication skills			Learning Skills			Social Inter. Skills			Overall PTPECS		
	*Sample*	*Mean*	*Med.*	*P*	*Mean*	*Med.*	*p.*	*Mean*	*Med.*	*p.*	*Mean*	*Med.*	*p*
*Years with PECS*				0.20			0.03*			0.91			0.21
1–5 Years 6 or more years	43 (59%) 30 (41%)	5.16 (0.55) 4.99 (0.60)	5.20 5.00		5.20 (0.57) 5.00 (0.45)	5.20 5.00		5.19 (0.49) 5.18 (0.38)	5.17 5.17		5.18 (0.48) 5.06 (0.43)	5.12 5.06	
Effect size													0.14
*Gender of child*				0.99			0.38			0.97			0.88
Boy Girl	41 (56%) 32 (44%)	5.11 (0.64) 5.06 (0.45)	5.00 5.00		5.17 (0.54) 5.05 (0.52)	5.00 5.00		5.20 (0.48) 5.17 (0.41)	5.17 5.17		5.13 (0.52) 5.09 (0.37)	5.12 5.06	
Effect size													0.14
*Frequency of using PECS*				0.44			0.64			0.71			0.91
Rarely Frequently	26 (36%) 47 (54%)	5.06 (0.76) 5.11 (0.44)	5.20 5.00		5.04 (0.68) 5.16 (0.42)	5.00 5.00		5.19 (0.52) 5.18 (0.40)	5.25 5.17		5.10 (0.61) 5.15 (0.37)	5.06 5.13	
Effect size													0.14

*P < .05; PECS = Picture Exchange Communication System; Med. = Median; PTPECS = Perception towards Picture exchange communication system scale; Social Inter. Skills = social interaction skills

A Kruskal-Wallis test was conducted to determine the association between three-level demographics and perception of the PECS (Table4). The results revealed a significant difference between the institution from which the children were receiving developmental services and the age the child began using the PECS. First, there was a significant difference between the institutions (inclusive school, special school, rehabilitation centre and early childhood centre) and social interaction skills, *χ*^*2*^ (3, n = 73) = 8.64, p = .04, with a large effect size of 0.49. The participants who indicated that their children were enrolled in an early intervention centre (*Md* = 5.83) had a higher median score than those whose children were enrolled elsewhere (inclusive school, *Md* = 5.17; special school, *Md* = 5.00; and rehabilitation centre, *Md* = 5.33).

**Table 4:** Summary of the results of the Kruskal-Wallis Test

N = 73		Communication Skills			Learning Skills			Social Inter. Skills			Overall PTPECS		
	*Sample*	*Mean*	*Med.*	*P*	*Mean*	*Med.*	*p.*	*Mean*	*Med.*	*p.*	*Mean*	*Med.*	*p*
***Category***				0.23			0.98			0.58			0.71
Mother Father Nanny	62 (85%) 6 (8%) 5 (7%)	5.11 (0.59) 4.77 (0.56) 5.20 (0.001)	5.00 5.00 5.20		5.12 (0.54) 5.07 (0.65) 5.08 (0.11)	5.00 5.20 5.20		5.21 (0.43) 5.02 (0.70) 5.13 (0.27)	5.17 4.83 5.33		5.15 (0.47) 4.95 (0.55) 5.14 (0.07)	5.09 5.06 5.19	
effect size													0.49
***Years caring for ASD***				0.16			0.07			0.19			0.30
1–5 years 6–10 years 11 or more years	28 (38%) 31 (43%) 14 (19%)	5.25 (0.60) 4.98 (0.60) 5.00 (0.39)	5.20 5.00 5.00		5.26 (0.65) 4.97 (0.45) 5.17 (0.34)	5.20 5.00 5.20		5.25 (0.53) 5.06 (0.38)	5.25 5.17 5.25		5.19 (0.55) 5.06 (1.08) 5.16 (0.32)	5.19 5.06 5.13	
effect size								5.28 (0.34)					0.49
***Institution***				0.07			0.29			0.04*			0.46
Inclusive school Special school Rehabilitation cent. Early entry. Cen.	27 (37%) 18 (25%) 14 (19%) 14 (19%)	5.07 (0.38) 5.00 (0.74) 4.88 (0.43) 5.46 (0.65)	5.00 5.00 5.00 5.80		5.05 (0.31) 5.11 (0.59) 5.00 (0.37) 5.37 (0.83)	5.00 5.00 5.00 6.00		5.11 (0.34) 5.06 (0.45) 5.16 (0.27) 5.51 (0.62)	5.17 5.00 5.33 5.83		5.08 (0.27) 5.06 (0.53) 5.02 (0.30)	5.06 5.06 5.13 5.88	
effect size											5.45 (0.67)		0.49
***Age of child***				0.82			0.54			0.15			0.94
1–5 years 6–10 years 11 or more years	8 (11%) 26 (36%) 39 (53%)	5.16 (0.91) 5.04 (0.73) 5.11 (0.35)	5.40 5.10 5.00		5.07 (1.02) 5.17 (0.54) 5.09 (0.39)	5.31 5.20 5.00		5.34 (0.76) 5.08 (0.49) 5.22 (0.32)	5.63 5.00 5.17		5.20 (0.85) 5.09 (0.53) 5.15 (0.29)	5.40 5.06 5.13	
effect size													0.49
***Age child began using PECS***				0.51			0.17			0.04*			0.05*
0–3 years 3–5 years 6 or more years	15 (20%) 35 (48%) 23 (32%)	5.22 (0.74) 5.01 (0.65) 5.12 (0.22)	5.00 5.00 5.00		5.31 (0.75) 5.01 (0.53)	5.20 5.00 5.20		5.39 (0.60) 5.08 (0.42) 5.21 (0.32)	5.33 5.00 5.17		5.31 (0.64) 5.04 (0.49)	5.13 5.00 5.04	
effect size					5.15 (0.27)						5.16 (0.19)		0.49

*P < .05; ASD = Autism Spectrum Disorder; PECS = Picture Exchange Communication System; Med. = Median; PTPECS = Perception towards Picture exchange communication system scale; Social Inter. Skills = social interaction skills; Early entry. Cen.= early intervention centre; Rehabilitation cent. = rehabilitation centre

Second, there was an association between the age the child began using the PECS, overall perception and social interaction skills. In relation to overall perception, there was a statistically significant difference between participants, *χ*^*2*^ (2, *n* = 73) = 5.82, *p* = .05, with a large effect size of 0.49. The median scores for those who indicated their children had used the PECS from before the age of three (*Md* = 5.13) was higher than those who noted that their children started using the PECS between 3 and 5 years of age (*Md* = 5.00) or when they were 6 years old or older (*Md* = 5.04).

There was also a significant difference between the age the child began using the PECS and the social interaction skills sub-scale, *χ*^*2*^ (2, 73) = 6.56, *p* = .04, with a large effect size of 0.49. The median scores showed that those who began using the PECS when they were 3 years or younger (*Md* = 5.33) indicated more improvement in social skills than those who were between the ages of 3 and 5 years (*Md* = 5.00) or 6 years and older (*Md* = 5.17).

### Association between measures

The association between communication, learning skills and social interaction skills was assessed using Spearman’s rho. The results revealed a moderate to large association between the sub-scales. There was a large correlation between communication skills and learning skills (r = .70, p = .001). Furthermore, there was a medium correlation between communication skills and social interaction skills (r = 44, p = .001). In addition, there was a medium correlation between learning support and social interaction skills (r = 45, p = .001).

## Discussion

Although there appears to be a relationship between individuals with ASD and a lack of language for communication, without language, individuals with ASD experience difficulty being understood and supported accordingly. Among the numerous interventions that enhance the communication of children with ASD, the PECS has been a beneficial AAC to promote the development of children with ASD [7]. Accordingly, the purpose of this study was to shed light on parents’ and caregivers’ perceptions of the impact that the PECS has on the domains of communication, learning and interacting with others. It is useful to indicate here that prior knowledge of participants about the impact of PECS on children with ASD was not documented before they participated in the training.

The results revealed that the participants noticed improvements in their children’s development. This finding concurs partly with other studies that demonstrated that the PECS contributes to enhanced communication, learning and social skills of children with ASD [28, 29] [26, 27, 31–37]. This finding is not surprising considering the amount of time that parents spend with their children. During any given day, parents likely spend more time with their children than any other person. Thus, their involvement in the rehabilitation process enables them to adhere to continual use and notice the gains associated with interventions, such as the PECS, that are provided to their children. This finding is noteworthy because there is currently a strong emphasis on inclusion in the UAE, thereby providing a conducive environment to helping children with ASD explore productive avenues in society. In addition, this finding may underscore the need for continual engagement and collaboration between specialists and parents in the usage of such interventions to facilitate the development of children with ASD.

The results revealed the association between communication, learning and social interaction skills. It is noteworthy that the intention was not to assume causality, but rather the strength of association between them. This finding concurs partly with previous studies that demonstrated that the use of the PECS enhanced communication skills in children with ASD [28, 29]. The results further revealed that, as the parents noted, the PECS had a positive impact on domains such as learning and social interactions with others. This finding was expected because communication is the essence of human interaction and daily living experiences. Communication is imperative for individuals to succeed in societal activities and thus should be developed. Almost half of the adults with ASD are unable to communicate, which has repercussions on their ability to learn and relate to other people in society. It is unsurprising that ASD has been linked to isolation because of sufferers’ inabilities to communicate, express their needs and be understood. It is recommended that policymakers in the UAE should advocate for continued research and development of the PECS in order to enable children with ASD to maximise the gains associated with its usage.

Early interventions appear to be important for enabling children with ASD to benefit from using the PECS [3]. There appears to be a consensus that providing early interventions can have a positive effect on the development of children with ASD [3, 8]. The results of this study revealed that parents who indicated that their children were enrolled at early childhood centres reported more enhanced social interaction skills when compared to those enrolled in regular schools and rehabilitation/special centres. Similarly, parents who noted that their children began using the PECS at the early age of under 3 years indicated they experienced more positive progress in social interaction and overall perception of the PECS than those who did not do so. This finding is not surprising because early access to rehabilitation services is synonymous with positive outcomes [8]. Since children with ASD are exposed to various interventions, these enable them to adapt and grow with such tools [3]. On the contrary, the late use of AAC could contribute to delayed development in areas such as learning and social interventions [3]. The parents of those who were exposed to the intervention noticed the positive effect of the PECS on communication. Therefore, policymakers should consider providing early access to the PECS intervention to children with ASD and their parents. This could help lessen the burden associated with ASD and ensure the children’s full inclusion in society.

Surprisingly, parents with fewer years of exposure to the PECS were more positive about learning support than their counterparts with more years of experience. The reasons are twofold. First, parents with fewer years of experience may have had younger children and identified early rehabilitation intervention as being central to their children’s development [3]. Thus, they may have been more acquainted with the PECS than their older counterparts. Furthermore, younger children learn more quickly and may adapt easily to using the PECS intervention daily. Second, parents with fewer years of experience may have invested more time and energy into studying new ideas or ways to use the PECS in order to support their children with ASD. Accordingly, the PECS yielded positive results related to their children’s learning abilities. However, it is recommended that future studies employ qualitative methods to afford an in-depth understanding of influence that years of experience have on parents’ perceptions of the PECS.

### Study limitations

It is noteworthy that the findings cannot be generalised. First, the schools provided the list of parents to the authors. Consequently, study bias may have occurred as the schools and rehabilitation centres may not have provided a complete list of all potential participants. However, we are of the opinion that the parents’ responses reflect the gains their children have achieved since they began using the PECS. Second, the data were collected from three of the seven Emirates in the UAE and thus may not be representative of the views of all parents with children with ASD. However, because the UAE has a shared culture, parents could receive common training in the PECS and share a common understanding of ASD. Since the parents had been trained to use the PECS, they may have provided appropriate responses to the items on the scale. As previously noted, the items were provided to the participants in both Arabic and English in order to ensure that they understood the statements and responded appropriately. Third, it was beyond this study’s scope to ascertain the influence of the level of severity of the children’s symptoms on their development. It is possible that the severity of disability could impact the parental experiences. Future studies could consider comparing the impact of PECS on child development based on their severity of ASD. Finally, the study did not measure the prior knowledge of parents before the intervention, which denied us the opportunity to ascertain the effectiveness of training intervention. Notwithstanding, it is recommended that a future intervention study be conducted in a similar non-western context in order to compare the impact of PECS training on the development of children with ASD. Nevertheless, to the best of our knowledge, the PTPECS is the first comprehensive instrument that deals with all of the useful developmental domains among most children with ASD.

## Conclusion and policy implications

The purpose of this study was to explore parents’ perceptions of the impact of the PECS among children with ASD in the UAE. While the PECS has been identified as an assistive tool to support the development of children with ASD, its effect has not been assessed in the UAE. The study’s findings revealed that the participants perceived the use of the PECS as favourable in supporting the development of children with ASD in the domains of communication, learning and social interaction skills. A moderate to large correlation was found between the measures. This may suggest that children with ASD who are exposed to the PECS may experience enhanced communication as well as appreciable development in other domains such as learning and social interaction skills. Moreover, other background variables, such as the institution at which their children are enrolled at, parents’ years of experience with the PECS, and the age the children began using the PECS, provided additional insight into the participants’ perceptions. The UAE government intends to facilitate the inclusion of all individuals with disabilities including those with ASD in societies. The findings of this study could provide useful guidelines to policymakers in their endeavours to realise equity and create a conducive environment that supports the development of all individuals in the UAE.

These findings have implications for policymaking and practice in the UAE. First, the findings emphasise the need for close collaboration between practitioners and parents. Since parents are able to identify progress their children made in relation to their exposure to the PECS and spend a considerable amount of time with their children, it is imperative to advocate parents’ continual usage of the PECS at home. Second, it is beneficial for educators and health providers to encourage early access to rehabilitation services. Early exposure could enable the children to gain exponentially from such an initiative. Third, there is a need for parents’ continual training and empowerment. In this instance, parents could be committed to learning and exploring avenues to support their children with ASD. Overall, everyone is capable of learning and, accordingly, parents’ commitment, through adherence to interventions at home, could have a positive effect on their children’s overall development.

## Data Availability

The datasets generated and/or analysed during the current study are not publicly available due ethical restrictions but are available from the corresponding author on reasonable request.
